# Up-regulation and clinical relevance of novel helicase homologue *DHX32 *in colorectal cancer

**DOI:** 10.1186/1756-9966-28-11

**Published:** 2009-01-22

**Authors:** Chunling Huang, Xianming Liang, Ruxin Huang, Zhongying Zhang

**Affiliations:** 1 Xiamen Center for Clinical Laboratory, Xiamen Zhongshan Hospital, Xiamen University, Xiamen 361004, PR China

## Abstract

**Background:**

This study aimed to find novel biomarkers for colorectal cancer.

**Methods:**

Fluorescent mRNA differential display PCR (DD-PCR) was used to screen the genes differentially expressed in colorectal cancer tissues and their adjacent tissues. The differentially expressed genes were confirmed by real-time PCR and then their clinical relevance (such as association with tumor location and lymph gland metastasis) was further investigated.

**Results:**

We identified by DD-PCR a novel RNA helicase, *DHX32*, which showed higher expression in colorectal cancer tissues than their adjacent tissues, and this result was confirmed by real time RT-PCR. In addition, we found that the level of *DHX32 *gene expression in colorectal cancer was significantly associated with cancer location, lymph gland metastasis, cancer nodal status, differentiation grade, and Dukes, stage.

**Conclusion:**

*DHX32 *may play an important role in the development of colorectal cancer and could serve as a novel biomarker for colorectal cancer after additional investigation.

## Background

Colorectal cancer (CRC) is one of the most common causes of cancer death throughout the world. Multistage development of the disease has been associated with remarkable genetic events, mainly at the level of oncogenes and oncosuppressor genes, most notably the adenomatous polyposis coli gene (APC) [1], ras [2,3], and p53 [4]. Although great advances have been made during the last few decades in understanding the molecular biology of colorectal cancer [5], the prognosis of patients with this neoplasm has not improved in parallel. The overall five-year survival rate remains poor (40–45%) [6]. It can be assumed that several genes involved in the pathogenesis of colorectal cancer are still unknown. Therefore, further elucidation of the molecular biology of colorectal cancer may be useful to devise new methods for the diagnosis and the treatment of the disease.

Several approaches have been taken to identify and compare gene expression in normal and disease states [7-11]. The differential display technique was employed in this study based on its ability to identify both up-regulated genes (putative oncogenes) and down-regulated genes (putative tumor/metastasis suppressor genes) simultaneously. Differential Display (DD) is a useful method to compare patterns of gene expression in RNA samples of different types or under different biological conditions [8,9]. The technique produces partial cDNA fragments by a combination of reverse transcription (RT) and PCR of randomly primed RNA. Changes in the expression level of genes are identified after separation of the cDNA fragments produced in an arbitrarily primed polymerase chain reaction on a sequencing-type gel. When combined with real-time quantitative PCR to eliminate false positives, DD becomes a powerful method for generating high confidence hits in the screening of hundreds of potentially differentially expressed transcripts. A number of genes such as UCC1 [12], Reg [13], and PIGR [14] have been detected by DD-PCR to be associated with colorectal cancer.

In this study, we found that *DHX32*, a novel RNA helicase, was significantly up-regulated in colorectal cancer compared to its adjacent normal tissue using a combination of DD-PCR and real-time PCR methods. Our results suggested that the level of *DHX32 *gene expression in colorectal cancer was significantly associated with cancer location, lymph gland metastasis, cancer nodal status, differentiation grade and Dukes' stage.

## Methods

### Subjects

34 pairs of specimens (tumor tissues and their adjacent normal tissues) and 14 tumor tissues were obtained from patients with colorectal cancer who underwent surgical resection at the Xiamen Zhongshan Hospital, Xiamen University in Xiamen during 2006 and 2007. The detail clinical and pathological characteristics of these 48 cases of samples were listed in a table 1. Adjacent normal tissues were defined as tissues which have no sign of cancer by visual inspection and which were located 3–5 cm surrounding the boundary of the cancer tissues. All of the patients gave informed consent prior to surgery. All specimens were reevaluated by a pathologist in the hospital. The specimens for assay were snap-frozen and stored in liquid nitrogen until analysis.

**Table 1:** Patients characteristics (n = 48)

	n (%)
Age (year)	
<59	25(52.1)
≥ 59	23(47.9)
	
Gender	
Male	22(45.8)
Female	26(54.2)
	
Tumor Location	
Colon	10(20.8)
Rectum	38(79.2)
	
Polypi	
+	14(29.2)
-	34(70.8)
	
Lymph metastases	
+	27(56.3)
-	21(43.7)
	
Tumor Nodal	
+	20(41.7)
-	28(58.3)
	
Tumor Differentiation	
Poor	9(18.8)
Median + WELL	39(81.2)
	
Dukes, Stage	
A+B	21(43.8)
C+D	27(56.2)

### RNA extraction and cDNA synthesis

Total RNA was prepared using Trizol reagent (Invitrogen, California, USA) according to the manufacturer's instructions. RNA was treated with DNase? (Invitrogen, California, USA) in the presence of 50 μM T7(dT12)AP2, T7(dT12)AP7 primer in 20 μl RT buffer (1×PCR buffer, 10 mM DTT, 0.25 mM dNTP), at 25°C for 5 minutes, followed by 42°C for 10 minutes and 50°C for 60 minutes. Reverse transcriptase was inactivated at 70°C for 15 minutes.

### Differential display

Differential display was performed using Hieroglyph mRNA Profile kit (Beckman, California, USA). Briefly, PCR amplification was done using 1.5 μl of the cDNA, primed with arbitrary P primer and anchored T primer. Amplification at (95°C 2 minutes) 1 cycle, (94°C for 15 seconds, 50°C for 60 seconds, 72°C for 2 minutes) 4 cycles, (94°C for 15 seconds, 60°C for 30 seconds, 72°C for 2 minutes) 25 cycles, followed by a final extension at 72°C for 7 minutes on a GeneAmp PCR system 9600 (Perkin-Elmer, Norwalk, USA). Following amplification of randomly primed mRNAs by RT-PCR, the cDNA products were heated at 94°C for 2 minutes and separated on a denaturing 5.6% polyacrylamide gel using a Genomyx LR DNA Sequencer (Beckman, California, USA). Bands exclusively present in either of two samples were considered as candidates of differentially expressed transcripts, which were excised, eluted, re-amplified, and subcloned into the pGEM-T easy vector (Promega, Madison, USA). The sequence reactions were performed by Invitrogen Corp (California, USA). Sequence homology to published database was analyzed with the BLAST program at the internet site of NCBI (National Center for Biotechnology Information) .

### Real-time quantitative reverse transcription polymerase chain reaction

We measured *DHX32 *expression in 48 tumor samples by real-time quantitative RT-PCR using TaqMan methodology in an ABI PRISM 7500 Sequence Detection System. The real-time RT-PCR allows, by means of fluorescence emission, the identification of the cycling point when PCR product is detectable. The Ct value inversely correlates with the starting quantity of target mRNA. Measurements were performed in duplicate and the controls were included in which the reaction mixture contained no cDNA. The amount of target mRNA after normalized to the endogenous reference *β*-actin was calculated by the Ct method as described by Liu W [15].

Primers and probes for *β*-actin and *DHX32 *mRNAs were chosen using the Primer Express 2.0 software (Applied Biosystems, Foster City, USA). The primers, placed in different exons, were designed to ensure that genomic DNA would not be amplified. Primer and probe nucleotide sequences for *DHX32 *(GenBank accession number NM_018180) were: DHX32-Fw 5'-GTCTTTCCATCCACTACCAGCAC-3', DHX32-Rev 5'-ATGATGACCCCATAGCT ACCCAA-3', and TaqMan probe 5'-(FAM) CGTGATATGCACACAGGTCCACAAG C (TAMRA)-3'. Primers and probe for *β*-actin mRNA were: *β*-actin-Fw 5'-TCACCCACACTGTGCCCATCTACGA-3', *β*-actin-Rev 5'-CAGCGGAACCGCTCATTGCCAATGG-3', and TaqMan probe 5'-(FAM)ATGCCC-X(TAMRA)-CCCCCATGCCATCCTGCGT-3'. Probes were purchased from Invitrogen Corp (Invitrogen, California, USA). The thermal cycling conditions were: 5 minutes at 95°C, followed by 60 cycles of 30 seconds at 95°C and 1 minute at 60°C.

### Statistic analysis

The Levene's test was performed to determine the homogeneity of variance for all the data, and then the paired Chi-Square test or 2-related samples Wilcoxon nonparametric test was performed to compare the positive rates and the levels of *DHX32 *gene expression between tumor tissue and its adjacent normal tissue in each patient, respectively. The Mann-Whitney U test was used for comparing the gene expression of *DHX32 *between the different groups according to various clinical and pathological variables. All of the statistical analyses were performed with SPSS 14.0 (Chicago, USA). A *P *value of less than 0.05 was considered to be statistically significant.

## Results

### Identification of a gene differentially expressed in the colorectal tumor and the adjacent normal tissue using DD-PCR

The modified DD-PCR method was used to identify genes uniquely and/or highly expressed in human CRC tissues by comparing with those in adjacent normal tissues. One cDNA band (size ranging between 700 bp and 800 bp) was found to be highly expressed in colorectal cancer tissues while barely expressed in matched adjacent normal tissues. The identified band was recovered, re-amplified, subcloned and sequenced. BLAST analysis of this nucleotide sequence revealed 99% homology to the gene *DHX32 *in the GenBank database.

### Confirmation of DHX32 differently expressed in colorectal tumors (CT) and their adjacent normal tissues (ANT) by real-time PCR

**Figure 1:**
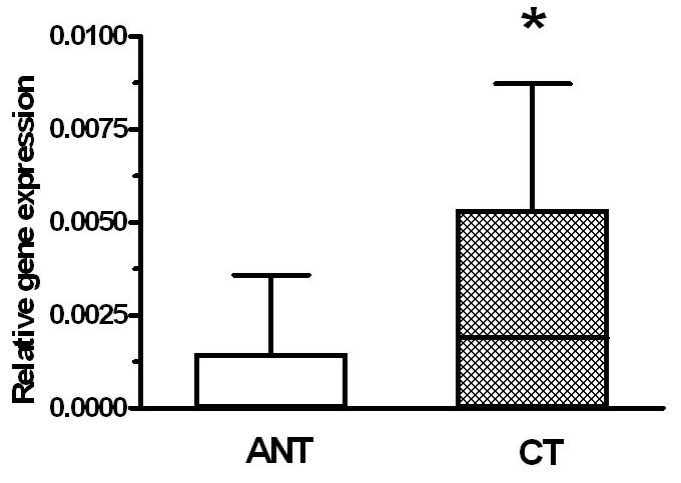
**Comparison of *DHX32 *gene expression between colorectal tumors (CT) and their adjacent normal tissues (ANT) *DHX32 *gene expression in colorectal tumors was significantly higher than that in their adjacent normal tissues**. Data were expressed with Box & Whiskers. ANT: Adjacent normal tissue; CT: Cancer tissues. *: *P *< 0.05.

We compared both positive rate and gene expression level in order to confirm the difference of *DHX32 *gene expression between colorectal tumors and their adjacent normal tissues. We found that the positive rate of *DHX32 *gene expression was significantly higher in the colorectal tumors (76.5%) than that in the adjacent normal tissues (26.4%) (Table 2). Consistent with the higher positive rate of *DHX32 *gene expression in the colorectal tumors, its gene expression level was also significantly higher than that in the adjacent normal tissues (Figure 1). In addition, the distribution of the patients with decreased, constant or increased gene expression of *DHX32 *was also analyzed. Whether it was decreased, constant or increased expression for each patient was arbitrarily classified according to the gene expression ratios between the tumor tissue and its adjacent normal tissue in each patient as described in the literature [16]: decreased expression (<0.8), constant expression (0.8~1.2), increased expression (>1.2). Tumor tissues from 58.8% of the patients displayed increased gene expression of *DHX32*. Those from 29.4% of the patients did not change and those from 11.8% of the patients even showed decreased gene expression of *DHX32 *(Table 3).

**Table 2:** The positive rate of *DHX32 *gene expression in the colorectal tumors and adjacent normal tissues

Group	DHX32 gene expression+		Positive rate
		
	+	-	
Tumor tissue	26	8	76.5% *
Adjacent normal tissue	9	25	26.4%

*: *P *< 0.01

**Table 3:** *DHX32 *gene expression in the colorectal tumors and their adjacent normal tissues

	Gene expression of DHX32 (CT/ANT, n = 34)		
	
	<0.8	0.8~1.2	>1.2
Patients	4 (11.8%)	10 (29.4%)	20 (58.8%)

1. CT (+) and ANT (-) treated as >1.2; 2. CT (-) and ANT (+) treated as <0.8; 3. CT (-) and ANT (-) treated as 1.

### Relationships between DHX32 gene expression and clinically pathological parameters

**Figure 2:**
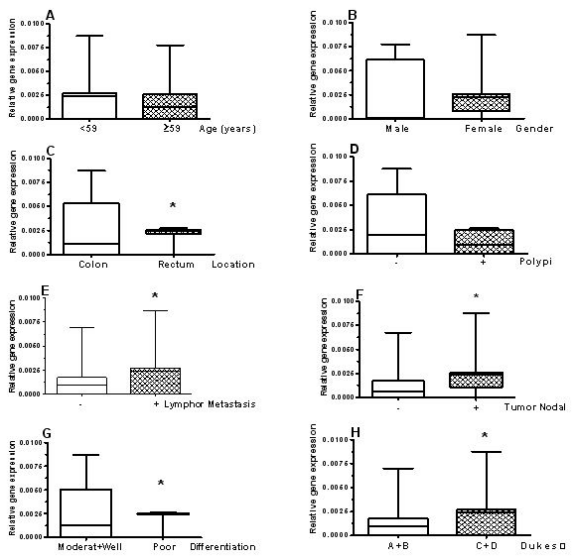
**The relationships between DHX32 gene expression and the clinical-pathological parameters (age, gender, tumor location, Polypi, lymph metastases, nodal status, differentiation grade and Dukes, stage) ***DHX32 *gene expression in colorectal carcinoma was not significantly associated with age **(A)**, gender **(B) **and Polypi **(D)**, but associated with tumor location **(C)**, lymph gland metastasis **(E)**, tumor nodal Status **(F)**, differentiation grade **(G) **and Dukes, stage **(H)**. Data were expressed with Box & Whiskers. *: *P *< 0.05.

In order to determine the relationships between *DHX32 *gene expression and the clinical-pathological parameters (age, gender, tumor location, Polypi, lymph metastases, nodal status, differentiation grade, and Dukes' stage), we compared the positive rate and the levels of *DHX32 *gene expression between the different groups according to various clinical and pathological variables. Although we did not observe significant differences of the positive rate of *DHX32 *gene expression between the groups according to each parameter (data not shown), our results suggested that the level of *DHX32 *gene expression in colorectal carcinoma was significantly associated with tumor location, lymph gland metastasis, tumor nodal status, differentiation grade and Dukes' stage (P < 0.05) (Figure 2). There were no apparent differences of *DHX32 *gene expression between the different groups classified by age, gender, and Polypi.

## Discussion

The study of the molecular biology of colorectal cancer has progressed rapidly, but the survival of patients with this neoplasm has improved rather modestly [17]. Consequently, further studies of CRC-related genes would help better understand the tumorigenesis of CRC and develop new methods for population screening, follow-up of treated patients, prognosis, and new therapies of the disease. In this study, we demonstrated that human *DHX32*, a novel RNA helicase, was up-regulated in colorectal cancer compared to its adjacent normal tissues. Furthermore, our results suggested that the level of *DHX32 *gene expression in colorectal carcinoma was significantly associated with tumor location, lymph gland metastasis, tumor nodal status, differentiation grade, and Dukes' stage.

*DHX32 *was originally identified as a novel RNA helicase with unique structure in the helicase domain, but with overall similarity to the DHX family of helicases [18]. RNA helicases are enzymes that utilize the energy derived from nucleotide triphosphate (NTP) hydrolysis to modulate the structure of RNA molecules and thus potentially influence all biochemical steps involving RNA which at least include transcription, splicing, transport, translation, decay, and ribosome biogenesis [19,20]. The involvement of RNA molecules in these steps is influenced by their tendency to form secondary structures and by their interaction with other RNA molecules and proteins [21]. *DHX32 *is composed of 12 exons spanning a 60-kb region at human chromosome 10q26 and encodes for a 743 amino acid protein with a predicted molecular weight of 84.4 kDa. *DHX32 *has a widespread tissue distribution and also has cross-species counterparts, such as 84 and 80% amino acid identity with mouse and rat counterparts, respectively. The high level of similarity between human and murine *DHX32 *and the widespread expression of *DHX32 *message suggest that it is an evolutionally conserved and functionally important gene.

With a few notable exceptions, the biochemical activities and biological roles of RNA helicases, including *DHX32*, are not very well characterized. In our study, we found that *DHX32 *was overexpressed in colorectal cancer compared with the adjacent normal tissues, suggesting that abnormal expression of *DHX32 *is associated with the development of colorectal cancer. The involvement of *DHX32 *in other cancer development was previously demonstrated by other groups. For example, the expression of *DHX32 *was dysregulated in several lymphoid malignancies [18,22]. DHX32 was reported as anti-sense to another gene, BCCIP (BRCA2 and CDKN1A Interacting Protein), and BCCIP was down-regulated in kidney tumors [23]. The overexpression of one of BCCIP isoforms can inhibit tumor growth [24].

So far, several groups have attempted to reveal the underlying mechanisms by which *DHX32 *involves in cancer development, but the exact biochemical activities and biological functions of *DHX32 *are still elusive. DHX32 contains sequences which are highly conserved between a subfamily of DEAH RNA helicases, including the yeast pre-mRNA splicing factor Prp43 [25], and its mammalian ortholog DHX15. The structural similarity of *DHX32 *to RNA helicases involved in mRNA splicing suggests a role in pre-mRNA splicing. It is possible that the dysregulation of the normal function of RNA helicases can potentially result in abnormal RNA processing with deleterious effects on the expression/function of key proteins in normal cell cycles and contribute to cancer development and/or progression. There are several examples of dyregulation of RNA helicases in cancer [26], mostly in the forms of overexpression including those of *DDX5 *[27] and *DDX6 *[28] which were reported to be overexpressed in colorectal tumor.

Our results indicated that the differential expression of *DHX32 *in colorectal carcinoma was significantly associated with tumor location, lymph gland metastasis, tumor nodal status, differentiation grade, and Dukes' stage. These results not only further confirmed the possible critical role of *DHX32 *in human colorectal development, but also suggested that additional studies may help develop *DHX32 *as a potential biomarker to judge the prognosis of colorectal cancer patients: the patients with higher gene expression of *DHX32 *may have worse prognosis.

In conclusion, to our knowledge, we are the first to report the more frequent and significant overexpression of human *DHX32 *in human CRC than that of the adjacent normal tissue, indicating that overexpression of *DHX32 *may play a pivotal role in the multistage carcinogenesis of human CRC. It still remains to be further investigated for the functions of *DHX32 *during the progression of colorectal cancer. *DHX32 *may also serve as a bio-marker for judging the levels of malignancy of colorectal cancer, which may guide the development of anticancer therapy regime after additional studies.

## Conclusion

*DHX32 *may play an important role in the development of colorectal cancer and additional studies may help use *DHX32 *as a novel biomarker for colorectal cancer.

## Competing interests

The authors declare that they have no financial competing interests.

## Authors' contributions

ZZ conceived of the study and guided the biochemical experiments.

CH performed DD-PCR and drafted the manuscript.

XL performed real-time PCR, analyzed data, collected tissue specimens and clinical records, and helped write the manuscript.

RH conceived of the idea and provided helpful comments.

All authors read and approved the final manuscript.
